# Large language model use in oral and maxillofacial surgery training: a national resident survey

**DOI:** 10.1007/s10006-026-01514-y

**Published:** 2026-02-21

**Authors:** Nolan Kranc, Edwin M. Rojas, Jacob Wise, Patrick Mansour, Gavin Lyell, Mena Morcos, Emerson A. Martins, Faisal A. Quereshy

**Affiliations:** 1 School of Dental Medicine, Case Western Reserve University, 9601 Chester Ave, Cleveland, OH 44106 USA; 2 School of Dentistry, University of Alabama at Birmingham, 1919 7th Ave S., Birmingham, AL 35233 USA; 3 Faculty of Medicine, University of Ottawa, Ottawa, ON Canada; 4 Department of Oral and Maxillofacial Surgery, University of Alabama at Birmingham, Birmingham, AL USA; 5 Department of Restorative Sciences, University of Alabama at Birmingham, Birmingham, AL USA; 6 Department of Oral and Maxillofacial Surgery, Case Western Reserve University, Cleveland, OH USA

**Keywords:** Artificial intelligence, Large language models, Oral and maxillofacial surgery, Education, Residency training, ChatGPT.

## Abstract

**Purpose:**

Large language models (LLMs) are advanced artificial intelligence (AI) tools capable of generating human-like text and are increasingly used in education, clinical care, and research. Little is known about their use within oral and maxillofacial surgery (OMFS) training. This study investigates LLM usage trends, perceived value, and educational integration among OMFS residents in the United States.

**Methods:**

A national, anonymous cross-sectional survey was distributed to OMFS residents via program directors. It gathered demographic data, LLM usage patterns, applications, perceived limitations, and attitudes toward incorporating LLMs into formal education.

**Results:**

Eighty-one residents responded, 79.0% (64/81) reported having used an LLM, and of that group, 96.9% (62/64) use ChatGPT. 51.9% (42/81) of respondents used LLMs at least monthly in residency; however, 97.5% (79/81) reported having received no formal LLM education during residency. Residents used LLMs for clinical decision support, board preparation, research, and career planning. Free-text responses revealed a wide spectrum of views. Some advocated for curricular integration and patient education applications, while others questioned the need for formal instruction. Some respondents supported integrating LLMs into curriculums and patient education while others questioned the need for formal instruction.

**Conclusion:**

LLMs are used frequently by OMFS residents for a variety of purposes. As AI and LLMs become embedded in healthcare, understanding how OMFS residents interact with LLMs is vital. These findings may guide curriculum development, fostering responsible and effective use of LLMs in surgical training and practice.

**Supplementary Information:**

The online version contains supplementary material available at 10.1007/s10006-026-01514-y.

## Introduction

Artificial intelligence (AI) is rapidly evolving, significantly impacting healthcare education. Among these advancements, large language models (LLMs) have emerged as powerful tools capable of comprehending and generating human-like text. Models such as ChatGPT have demonstrated potential in enhancing medical education through personalized feedback, case scenario generation, and content creation [1, 2]. For instance, research has investigated the knowledge, attitudes, and practices of undergraduate medical students regarding LLM utilization, highlighting the potential of these models to enhance learning experiences [3, 4]. In dental education, LLMs have been discussed for their ability to provide personalized feedback and generate educational content, contributing to improved educational outcomes [5–7].

A study surveying internal medicine residents found that 56% reported the use of LLMs in a clinical setting, 94% of participants envisioned a future in integrating LLMs, and none of the participants received any formal guidance [8]. Another study monitoring the use of LLMs by radiology residents showed that there was significant agreement between LLM-assisted reports and clinical reports by board-certified radiologists [9]. In addition, several studies focusing on plastic surgery residency programs suggested that LLMs could serve as valuable teaching assistants, providing interactive case studies and simulations [10–12]. These findings underscore the potential benefits of integrating LLMs into healthcare training, including enhanced clinical decision-making and personalized learning pathways.

Despite the increase in presence of LLMs in residency training, there are no studies which evaluate their use within oral and maxillofacial surgery (OMFS) residency programs [13–15]. Understanding resident engagement with these tools is essential, as LLMs may enhance clinical decision-making, research capabilities, and educational experiences [16, 17]. We hypothesize that OMFS residents are actively using LLMs for a variety of educational, clinical, and research purposes, and that its usage is likely to increase because of the surge of AI in OMFS [13, 18]. Accordingly, there is a growing need for formal guidance on the appropriate integration of LLMs into residency training [19]. This study aims to investigate current trends in LLM usage and education among OMFS residents in the United States, offering insight that may inform the future integration of AI into surgical training.

## Materials and methods

### Survey design and distribution

A 16-question survey was developed to evaluate OMFS residents’ knowledge, attitudes, and practices regarding LLMs. The survey included multiple-choice, multi-select, and open-ended questions, and was created using REDCap, a secure online data capture tool accessed by Case Western Reserve University. The survey was emailed to all OMFS residency program directors across the United States, with a request to share the link with their residents. There are approximately 1200 current OMFS residents in the United States.

### Variables

Covariates included gender, post-graduate year, program type (single or dual-degree), type of LLM platform (ChatGPT, BingAI, Gemini, BERT, other, or none), frequency of using LLMs for OMFS learning or practice (daily, once a week, once a month, multiple times a month, or none), and frequency of most used application of LLMs (clinical situation, self-study and learning, research, career related, other, or none). “Clinical situations” included patient interviews, physical exams, diagnosis assistance, procedure explanation, medication dosage, and admission/discharge summary. “Self-study and lecture” included explaining a concept, creating practice questions, and answering a scientific or clinical question. “Research” included aid with writing a manuscript, writing institutional review board submissions, communication with supervisors, data analysis, and literature search. “Career related” included resume/CV writing, cover letter writing, and making a presentation.

### Data collection, analyses, and ethical considerations

Institutional Review Board (IRB) exemption by Case Western Reserve University was obtained on 5/28/24 prior to study commencement (IRB #STUDY20240613). Participation was voluntary and anonymous. No identifying information was collected. Data was exported from RedCap into a secure Google spreadsheet available to the authors in this study. Data was tabulated using Microsoft Word and analyzed using GraphPad Prism 10.5.0.

## Results

### Demographics

Covariates of participant demographics were recorded in Table 1. Approximately 6.8% of OMFS residents (81/~1200) in the United States responded to the survey. There were 2.3-fold more male (66.7%, 54/81) than female participants (29.6%, 24/81). No participants responded as non-binary/other and 3.7% (3/81) participants preferred not to state their gender. Survey responses were slightly higher (1.3-fold) for residents in 4-year (56.8%, 46/81) compared to 6-year (43.2%, 35/81) OMFS programs. Survey responses decreased progressively as postgraduate years advanced. Although only 2 respondents had formal education in computer science, 79.0% (64/81) of participants reported using a LLM before. Among LLM users, nearly all (96.8%, 62/64) reported use of ChatGPT while only 24 (29.6%) respondents reported use of additional LLMs. 17 participants have not used a LLM during OMFS residency training.

**Table 1:** Summary of covariates for the survey responses from the OMFS residents (*n* = 81). Covariates included gender, residency program length, post-graduate year (PGY), academic background with computer science, experience with large Language models (LLMs), and if participants used LLMs during residency training. *Percentages were calculated from (n/81) x 100%. 95% confidence intervals (CI) were calculated from the Wilson/Brown method using GraphPad Prism 10.5.0

Participants	*n*	%	95% CI
	81	–	–
Gender			
Male	54	66.7	55.9–76.0
Female	24	29.6	20.8–40.3
Non-binary/other	0	0	0.0–4.5
Prefer not to say	3	3.7	1.0–10.3
Residency program length			
4-year	46	56.8	45.9–67.0
6-year	35	43.2	33.0–54.1
Post-Graduate Year (PGY)			
1	26	32.1	22.9–42.9
2	21	25.9	17.6–36.4
3	16	19.8	12.5–29.7
4	15	18.5	11.6–28.3
5	2	2.5	0.4–8.6
6	1	1.2	0.1–6.7
Academic background with computer science			
Yes	2	2.5	0.4–8.6
No	79	97.5	91.4–99.6
Experience with Large Language Models (LLM)			
Yes	64	79.0	68.9–86.5
No	17	21.0	13.5–31.1
LLMs used during residency training*			
ChatGPT	62	76.5	50.5–69.1
BingAI	8	9.9	4.0–14.6
Gemini	7	8.6	3.3–13.4
BERT	1	1.2	5.0e^− 2^ – 5.3
Other	8	9.9	4.0–14.6
None	17	21.0	10.6–24.9

### Frequency and usage

Approximately half (51.9%, 42/81) of respondents reported using a LLM at least once per month. Despite this frequency, 97.5% (79/81) reported receiving no formal education or guidance on the use of LLMs during their residency training. Reasons for usage of LLMs varied among residents including clinical decision support, board preparation, research, and career planning. Of LLM users, the most common use of LLMs was for self-study and/or lecture purposes (38.3%, 31/81). Additionally, 9.9% (8/81) of participants reported using LLMs for career-related questions/planning and 7.4% (6/81) for clinical contexts (Table 2).

**Table 2:** Survey responses describing the frequency and context of using LLMs in OMFS residency training. 95% confidence intervals (CI) were calculated from the Wilson/Brown method using GraphPad Prism 10.5.0

	*n*	%	95% CI
In the past year of your residency training, how often have you used a Large Language Model for OMFS learning or practice?			
Daily	7	8.6	4.2–16.8
Once per week	5	6.2	2.7–13.6
Once per month	20	24.7	16.6–35.1
Multiple times per month	10	12.3	6.8–21.3
Never	39	48.2	37.6–58.9
For which of the options do you use LLMs most frequently? Choose one.			
Clinical	6	7.4	3.4–15.2
Self-study/lecture	31	38.3	28.4–49.2
Research	3	3.7	1.0–10.3
Career-related	8	9.9	5.1–18.3
Other	1	1.2	0.1–6.7
None	32	39.5	29.6–50.4

### Opinions and ethical considerations

48.1% (39/81) of those who used LLMs either agreed or strongly agreed that explanations of OMFS concepts by LLMs were generally accurate. 76.5% (62/81) of respondents considered it ethical to use LLMs for explanations of OMFS topics. Similarly, 79.0% (64/81) considered it ethical to use LLMs for resumes and cover letters. In contrast, ethical opinions on using LLMs for academic manuscripts were divided with 50.6% (41/81) who viewed it as ethical, while 49.4% (40/81) either disagreed or strongly disagreed (Table 3).

**Table 3:** Summary of survey responses describing the guidance and ethical considerations of LLMs during OMFS residency training. 95% confidence intervals (CI) were calculated from the Wilson/Brown method using GraphPad Prism 10.5.0

	*n*	%	95% CI
Have you received any guidance from your residency program regarding the optimal use of artificial intelligence or large language models in your residency program?			
Yes	2	2.5	0.4–8.6
No	79	97.5	91.4–99.6
Explanations of oral surgery concepts provided by large language models are generally accurate.			
Strongly Agree	3	3.7	1.0–10.3
Agree	36	44.4	34.1–55.3
Disagree	5	6.2	2.7–13.6
Strongly Disagree	2	2.5	0.4–8.6
No experience	35	43.2	33.0–54.1
Using large language models in the context of an oral surgery concept being explained to me is ethical.			
Strongly Agree	16	19.7	12.5–29.7
Agree	46	56.8	45.9–67.0
Disagree	15	18.5	11.6–28.3
Strongly Disagree	2	2.5	0.4–8.6
No experience	2	2.5	0.4–8.6
Using large language models in the context of writing resumes or cover letters is ethical.			
Strongly Agree	14	17.3	10.6–26.9
Agree	50	61.7	50.8–71.6
Disagree	14	17.3	10.6–26.9
Strongly Disagree	3	3.7	1.0–10.3
No experience	0	0	0.0–4.5
Using large language models in the context of writing academic manuscripts is ethical.			
Strongly Agree	7	8.6	4.2–16.8
Agree	34	42.0	31.8–52.8
Disagree	29	35.8	26.2–46.7
Strongly Disagree	11	13.6	7.8–22.7
No experience	0	0	0.0–4.5

Open ended questions revealed a variety of opinions on the usage of LLMs. Some respondents advocated for the integration of LLMs into educational curricula and patient education (SI Table 1), whereas others expressed skepticism regarding the necessity of formal instruction (SI Table 2). One respondent envisioned future systems generating clinical notes and standardized patient explanations (SI Table 3, Entry 3). Another respondent described LLMs as valuable for forming differential diagnoses (SI Table 1, Entry 28). Although several participants questioned the accuracy of LLMs when used for clinical and surgical information (SI Table 2), several recognized its potential in forming differential diagnoses, aid in summarizing large amounts of information, or gathering quick, succinct information on a variety of medical topics (SI Table 1).

## Discussion

This cross-sectional study used a 16-question survey to glean how OMFS residents in the United States currently perceive and engage with LLMs in their training. A high yield of male responses was not surprising since there is still a higher percentage of male OMFS residents and practicing clinicians compared to other genders [20, 21]. Most responses were from OMFS residents during their early post-graduate years of training, with LLMs more frequently used for self-study/lecture. This may be attributed to the intense didactic and clinical training within the first two years of residency, especially when residents prepare to take the USMLE Step 1 Exam [22]. It also may be attributed to lower year residents being less fatigued from residency and being more willing to respond to surveys sent to them. With the explosion of research of its applications in education, LLMs are currently being explored for their potential to generate questions from textbooks, which could be a powerful study tool [23].

Although many respondents did not have formal training in computer science, the majority of residents reported exposure to LLMs throughout their career training, the dominating platform being ChatGPT [24]. ChatGPT is the most popular conversation LLM used in healthcare [25]. ChatGPT has shown to have high accuracy with multiple-choice questions in topics of OMFS, yet still struggles to solve complex cases, such as orthognathic surgery and oral oncology [26]. Although LLMs are widely used in OMFS for research, patient education, and learning, there is a pressing need for more oral and maxillofacial conditions and diseases to be included and elaborated upon [24, 27, 28].

About 50% of our respondents currently use LLMs in their OMFS training at least once a month, which is about double the percentage of internal medicine residents (26%, 40/152) from a similar study [8]. Both studies agree that residents are open to learning more about how to incorporate LLMs within their training given they are provided with a nurturing atmosphere for exploring their applications and potential.

Most respondents received no guidance for using LLMs in their OMFS training. In a study surveying OMFS residents to glean barriers to their research productivity, most respondents (79%,115/145) stated that they do not receive formal research training or courses [29]. It seems that OMFS residents are not adequately equipped with tools that can help aid their research training, one of them being LLMs. This observation could be because of an OMFS program’s allocation of resources and faculty’s willingness to use LLMs. Some respondents in our study stated that LLMs are still not accepted by some of the previous generations of oral and maxillofacial surgery attending faculty, which may limit its use and appreciation while being a resident.

Although most respondents believed that it is ethical to use LLMs for explaining concepts related to OMFS, 21.0% of respondents disagreed. The same trend was observed for its ethicality in writing resumes/CVs and cover letters. Many responses described issues concerning accuracy, reliability, generalization, and lack of incorporation by many oral and maxillofacial surgeons, particularly in academic settings, likely because of how rapidly AI tools evolve and its ambiguous perception within the scientific and clinical community. In a cross-sectional study surveying medical students across the United States, participants mentioned that LLMs such as ChatGPT at times showed bias and lacked accuracy when searching for diagnoses and medical concepts [30].

Interestingly, nearly the same number of responses considered and did not consider it ethical to use LLMs for writing academic manuscripts, potentially reflecting a neutral stance toward the idea. Academic writing can be challenging for many clinicians and researchers, especially without formal writing experience or having language barriers, undermining their ability to engage in scientific communication. LLMs can help aid in the writing and editing of scholarly works when used appropriately while also promoting transparency and supporting diversity and inclusion within OMFS research [31]. However, concerns have been raised regarding the ethical use of AI in medicine, the potential for biased training data, and the need for responsible implementation to avoid issues such as AI-generated plagiarism [32]. Addressing these challenges requires the development of comprehensive guidelines and frameworks to ensure the ethical and effective use of LLMs in medical and surgical education [33].

The study’s response rate was currently limited, with 81 responses received. Obtaining survey participation from OMFS residents poses a significant challenge, as their demanding clinical schedules, limited availability, and high levels of stress or anxiety can hinder engagement with voluntary research efforts [34]. Furthermore, the reliance on self-reported data may introduce social desirability bias, with respondents potentially underreporting or overreporting their use of LLMs due to perceived ethical considerations or stigma associated with AI usage in professional settings. Response rates for surveys distributed to OMFS residents at the national level are generally much lower, particularly electronically, than when distributed within a single institution by paper format [35]. Although a study reports that OMFS residents may be overwhelmed with completing surveys [36], it is still essential to learn about the experiences OMFS residents face and obtain their feedback in order to improve the educational tools within their training.

In summary, this study provides foundational insight into how OMFS residents in the United States are engaging with LLMs, identifying current usage patterns, perceived benefits, and barriers to adoption. As AI continues to permeate healthcare education, understanding the role of large language models in residency training becomes increasingly important. This study informs the development of evidence-based recommendations for incorporating LLMs into OMFS education. With thoughtful integration, these tools have the potential to enhance clinical reasoning, streamline academic workflows, and improve overall resident learning experiences. Future work should explore structured training on LLM use and establish best practices to ensure their ethical and effective deployment in surgical education.

## Supplementary Information

Below is the link to the electronic supplementary material.

ESM 1 (DOCX 31.2 KB)

## Data Availability

No datasets were generated or analysed during the current study.
