# Supplementary material for: Large language model use in oral and maxillofacial surgery training: a national resident survey
Source: Oral Maxillofac Surg. 2026 Feb 21;30(1):32. doi: 10.1007/s10006-026-01514-y (PMC12923429; doi:10.1007/s10006-026-01514-y)
Supplement: Supplementary file 1 — (DOCX 31.2 KB) [file 10006_2026_1514_MOESM1_ESM.docx]

**Large Language Model Use in Oral and Maxillofacial Surgery Training: A National Resident Survey**

**Journal:** *Oral and Maxillofacial Surgery*

Nolan Kranc, BSc,^1^ Edwin M. Rojas, PhD,^2^ Jacob Wise, BSc,^3^ Patrick Mansour, BS,^4^ Gavin Lyell, BS,^5^ Mena Morcos, DMD,^6^ Emerson A. Martins, DDS, MSc, PhD,^7^ and Faisal A. Quereshy, MD, DDS, MBA, FACS^8^

^1^Dental student, School of Dental Medicine, Case Western Reserve University, Cleveland, OH, United States.

^2^Dental student, School of Dentistry, University of Alabama at Birmingham, Birmingham, AL, United States.

^3^Medical student, Faculty of Medicine, University of Ottawa, Ottawa, ON, Canada.

^4^Dental student, School of Dental Medicine, Case Western Reserve University, Cleveland, OH, United States.

^5^Dental student, School of Dentistry, University of Alabama at Birmingham, Birmingham, AL, United States.

^6^Resident, Department of Oral and Maxillofacial Surgery, University of Alabama at Birmingham, Birmingham, AL, United States.

^7^Assistant Professor, Department of Restorative Sciences, University of Alabama at Birmingham, Birmingham, AL, United States.

^8^Professor, Department of Oral and Maxillofacial Surgery, Case Western Reserve University, Cleveland, OH, United States.

**Address correspondence and reprint requests to the following co-corresponding authors:**

**Nolan Kranc:** School of Dental Medicine, Case Western Reserve University, Cleveland, OH, United States. **Email:** [nek46@case.edu](mailto:nek46@case.edu)

**Edwin M. Rojas:** School of Dentistry, University of Alabama at Birmingham, Birmingham, AL, United States. **Email:** [emrojas@uab.edu](mailto:emrojas@uab.edu)

**Supplementary Information**

| **Entry** | **If you have used large language models in OMFS training, what benefits do you think arise from having this tool in residency?** |
| --- | --- |
| 1 | I have used this tech for chart review |
| 2 | Helps with job inquiries, rejections of positions, acceptances. Also, helped write a recommendation letter for a friend. |
| 3 | It's an incredible tool for learning very quickly. This can be applied to literally everything medicine. |
| 4 | Saves time. |
| 5 | It provides the benefit of being able to organize and synthesize the data to a focused question. |
| 6 | Quickly digesting manuscript information. Using. Speechify to read while commuting |
| 7 | Putting in a textbook, then asking AI to answer a question. Easier than searching in text. |
| 8 | I have not used them in residency, I think it could benefit residents in quick search of topics especially for last second OR prep, but I do not always trust the validity given that it can pull information from the entire internet including satirical articles and information that is not factual |
| 9 | For research writing (proposals, abstracts) this is a simple way to proofread. Can help with sentence clarity or rearranging a paragraph. |
| 10 | Immediate access to simple explanations of complex topics |
| 11 | I believe that language models could be beneficial in reducing the monotony of charting if they were able to be integrated into the EHR process. I still think that they are not an accurate source of information and need to be fact checked/vetted with other resources (uptodate, lexicomp, primary literature, etc) before implementing anything into clinical care; same as providers who Google medical questions and trust that answer without confirming. I think not doing so is unethical and shorting patients on quality care. I have used chat gpt to generate differential diagnoses as it is good at finding esoteric disease processes that the clinician can then further investigate. |
| 12 | Ability to gather large amounts of data and summarize into concise amount of information. |
| 13 | It allows for a different way to investigate issues, and come up with ideas that you may not otherwise |
| 14 | Summaries of topics, writing letters to insurance companies |
| 15 | All that research engines and books can provide if incorporated into the model |
| 16 | For guidance |
| 17 | For secretarial work, this helps optimize time and be more efficient |
| 18 | It can answer questions that are not easily googleable, and it can guide you in a direction to further read publications on. AMBOSS GPT is helpful for dosing and medical questions. |
| 19 | Easy access to concise information on topics. Good starting point to dive deeper. Helpful in figuring out potential first steps for new patients |
| 20 | Synthesizing information and studies into concise recommendations. |
| 21 | Quick and easy |
| 22 | Efficiency |
| 23 | Problem solving complex clinical situations once language models have developed |
| 24 | Increasing efficiency and rate of understanding complex cases |
| 25 | Fast way to learn. Also has great detailed patient education. |
| 26 | Efficiency |
| 27 | Clinical practice guidelines |
| 28 | Great learning tool Good aid for forming differential diagnoses |
| 29 | Rapid scanning of the literature for highlights. Answering questions and providing sources. |
| 30 | Speed & allowance of focus on higher level learning |
| 31 | It can make it easier to find information on a specific topic |
| 32 | Little benefit |
| 33 | Readily accessible tool for quick answers |
| 34 | Ability to get information for specific questions or scenarios using peer reviewed sources or textbooks that may take a long time otherwise |
| 35 | Receiving a quick and succinct answer for clinical subjects/medical concepts. |
| 36 | Very useful for creating templates and scripts for letters for consults to give to patients, and written instructions to patients. It also aids in helping to search for journal articles. |

**Supplementary Table 1.** Optional responses (36 out of 81) to the open-ended question: “If you have used large language models in OMFS training, what benefits do you think arise from having this tool in residency?”

| **Entry** | **If you have used large language models in OMFS training, what issues have you encountered with using this tool in residency?** |
| --- | --- |
| 1 | The language used is somewhat long winded and not always accurate. But does help and give a starting point. Can't just copy and paste what is written, but is a good starting place. |
| 2 | It's level of detail. You can get the AI to eventually explain everything on a deeper level that only a physician or someone with schooling or extensive self research would know, but it takes creative wording which is annoying. |
| 3 | Not everything is accurate. I don't take everything as truth and I double check its accuracy as opposed to relying on it for my sole source of information. |
| 4 | Some times the information provided lacks depth and is superficial |
| 5 | Fills in some gaps with information that might not be true. |
| 6 | One of our residents got in trouble for using an LLM to write his notes - there was concern that it was poor patient care and a HIPAA violation |
| 7 | Does not consider the nuances of clinical practice. Our job is very human centered and a lot can play into that. |
| 8 | As I said above, trusting the validity of the source is always in question. |
| 9 | AI tends towards basic summaries - so far Chat GPT doesn't seem to understand nuance very well beyond blanket statements. |
| 10 | The information is generalized, whereas patient specifics are highly individualized |
| 11 | Accuracy Authenticity Ethics |
| 12 | Sometimes not relevant answers |
| 13 | Accuracy, hallucinating |
| 14 | Incorrect information when asked specific details |
| 15 | Inability to cite, inability to fact check |
| 16 | Inaccuracies |
| 17 | You need to be careful using it as a knowledge resource and fact check it |
| 18 | Very inaccurate, unable to find esoteric information |
| 19 | Some topics only have more superficial and generic information available. |
| 20 | Sometimes inaccurate. |
| 21 | Not specific enough answers |
| 22 | Inaccuracy or not understanding concept being asked |
| 23 | Inaccuracies and data that is generalised |
| 24 | AI models refusing to provide information on regarding healthcare advice |
| 25 | The depth of the knowledge |
| 26 | Not specialty specific information |
| 27 | Isn't always accurate. Requires reviewing. |
| 28 | Difficulty verifying accuracy for questions I don't already know the answer to |
| 29 | Reference accuracy |
| 30 | Its not 100% accurate |
| 31 | Accuracy of information |
| 32 | Not always accurate, does not always understand specific OMFS concepts |
| 33 | It seems OMFS can be too "niche" and may not provide the information exactly that you are seeking |
| 34 | Answers are sometimes vague or inaccurate. Some questions require further independent research. |
| 35 | I've attempted to use it for notes and documentation and find it limited. I usually need to rewrite the prompt or the note. Additionally, some information can actually be incorrect, so finding a model that uses reputable sources is necessary. It's better to learn/understand the concept through textbooks and articles, than apply AI as a primary learning tool. |

**Supplemental Table 2.** Optional responses (35 out of 81) to the open-ended question: “If you have used large language models in OMFS training, what issues have you encountered with using this tool in residency?”

| **Entry** | **How can OMFS programs best support residents on the learning and application of large language models?** |
| --- | --- |
| 1 | It would be interesting to implement it |
| 2 | They should probably educate themselves, then we can talk about it at an in-service meeting. |
| 3 | Honestly, I want AI to basically write all of my clinical notes as well as provide patients with base level knowledge they should know about the procedure they are having completed. |
| 4 | It seems like a personal preference and doesn't need program involvement. |
| 5 | I think provided sound ethical clinical situations where the information can be used. |
| 6 | Formalizing strategies to maximize output. |
| 7 | Efficiency of documentation |
| 8 | I think just familiarizing yourself with what is out there is the best thing we can do. |
| 9 | For quick references, being able to search "OKC" for example and getting a brief summary on the main points. Having a language model that is specific to OMFS would be great. As a learner, sometimes researching certain topics can give you textbook answers but sometimes you need real world applications or actual surgical references on how the surgery is completed. Most textbooks I use for reference can always give me information on a topic, but it feels that virtually none of them will walk you through how a surgery is typically completed. |
| 10 | Filtering through poor evidence |
| 11 | I don't have ideas on this since I haven't even thought that many other people were using these tools |
| 12 | Supporting/Developing/Contributing to language models specific to our craft |
| 13 | Unsure as this is an evolving resource. |
| 14 | Educate residents and attendings regarding possible applications and how to utilize in residency. |
| 15 | Present good ways it can be used, good tools such as OpenEvidence |
| 16 | Lectures and describe how and when it should be used |
| 17 | Too unfamiliar with the models to comment |
| 18 | Videos and modules on how to use, literature on specific applications. |
| 19 | More information on the topic |
| 20 | I don't think these models are developed enough for safe, ethical, or efficient use in training yet. |
| 21 | I do not support it |
| 22 | Understanding where it can be used to help provide efficiency. |
| 23 | The program would need someone who is well versed in them and can provide instruction on their basic use, whether that person be a resident or attending. There is also an issue regarding things like private health information and HIPPAA compliance that once would have to ensure are being done appropriately. |
| 24 | Have outside lecturers in OMFS come and speak about how the effectively use AI for efficiency |
| 25 | I don't think much, we are already a fairly tech savvy generation. |
| 26 | No thoughts |
| 27 | Integration in Epic, for ease of use. |
| 28 | Attendings who use it themselves. Also upper level residents teaching lower level residents how to use. |
| 29 | Programs can advise residents how to best utilize large language models. |
| 30 | Help determine where language models are appropriate and clear up what is considered ethical and not |
| 31 | incorporate into EMR programs and clinical software for use and to gain experience in using AI Language Models |
| 32 | Do more education on this |
| 33 | Gotta use it lol |
| 34 | Encourage use. Explore ambient listening models to reduce admin and documentation burden |
| 35 | Guidance on how to interpret and use the information (ie, the machine has never physically performed any procedure itself). |
| 36 | Use them more in every way possible and evaluating the efficacy of the results to learn how to revise & modify the prompts for more effective outputs |
| 37 | Integrate it into daily use |
| 38 | Unsure |
| 39 | Understanding its role and highlighting the benefits of learning while using peer reviewed sources and textbooks. A workshop could potentially be helpful |
| 40 | Explain how the proper use of AI can help guide clinical education. Programs can also try utilizing AI for early boards review. |
| 41 | For now, I think it can be best used to dictate physician to physician letters for clearance/risk-stratification, or to create pre-operative/post-operative instructions for patients. It is also useful for organizing presentations and citing sources as necessary. Although it can be used to help search for potential searches as well. |

**Supplemental Table 3.** Optional responses (41 out of 81) to the open-ended question: “How can OMFS programs best support residents on the learning and application of large language models?”
